# High Variability in Oral Glucose Tolerance among 1,128 Patients with Cystic Fibrosis: A Multicenter Screening Study

**DOI:** 10.1371/journal.pone.0112578

**Published:** 2014-11-13

**Authors:** Nicole Scheuing, Reinhard W. Holl, Gerd Dockter, Julia M. Hermann, Sibylle Junge, Cordula Koerner-Rettberg, Lutz Naehrlich, Christina Smaczny, Doris Staab, Gabriela Thalhammer, Silke van Koningsbruggen-Rietschel, Manfred Ballmann

**Affiliations:** 1 Institute of Epidemiology and Medical Biometry, Central Institute for Biomedical Technology, University of Ulm, Ulm, Germany; 2 Cystic Fibrosis Centre, Saarland University Hospital for Pediatric and Adolescent Medicine, Homburg/Saar, Germany; 3 Clinic for Pediatric Pneumology and Neonatology, Hannover Medical School, Hannover, Germany; 4 Department of Pediatric Pulmonology, St. Josef Hospital Pediatric Clinic, Ruhr University Bochum, Bochum, Germany; 5 Department of Pediatrics, Justus-Liebig University Giessen, Giessen, Germany; 6 Medical Clinic I, Pneumology and Allergology, University Hospital Frankfurt/Main, Goethe University, Frankfurt/Main, Germany; 7 Division of Pulmonology and Immunology, Department of Pediatrics, Charité Berlin, Berlin, Germany; 8 Department for Pediatric Pulmonology and Allergology, Medical University of Graz, Graz, Austria; 9 Cystic Fibrosis Centre Cologne, Childrens Hospital, University of Cologne, Cologne, Germany; University of Tübingen, Germany

## Abstract

**Background:**

In cystic fibrosis, highly variable glucose tolerance is suspected. However, no study provided within-patient coefficients of variation. The main objective of this short report was to evaluate within-patient variability of oral glucose tolerance.

**Methods:**

In total, 4,643 standardized oral glucose tolerance tests of 1,128 cystic fibrosis patients (median age at first test: 15.5 [11.5; 21.5] years, 48.8% females) were studied. Patients included were clinically stable, non-pregnant, and had at least two oral glucose tolerance tests, with no prior lung transplantation or systemic steroid therapy. Transition frequency from any one test to the subsequent test was analyzed and within-patient coefficients of variation were calculated for fasting and two hour blood glucose values. All statistical analysis was implemented with SAS 9.4.

**Results:**

A diabetic glucose tolerance was confirmed in 41.2% by the subsequent test. A regression to normal glucose tolerance at the subsequent test was observed in 21.7% and to impaired fasting glucose, impaired glucose tolerance or both in 15.2%, 12.0% or 9.9%. The average within-patient coefficient of variation for fasting blood glucose was 11.1% and for two hour blood glucose 25.3%.

**Conclusion:**

In the cystic fibrosis patients studied, a highly variable glucose tolerance was observed. Compared to the general population, variability of two hour blood glucose was 1.5 to 1.8-fold higher.

## Introduction

Cystic fibrosis (CF) is a life-limiting autosomal recessive illness occurring in about one of 2,500 newborns in Europe. As the life expectancy for CF patients has increased due to earlier diagnosis and improved care, CF-related comorbidities became more frequent over the last decades. A growing comorbidity with impact on the course of CF is cystic fibrosis-related diabetes (CFRD). The latest comprehensive guidelines for CFRD recommend yearly screening for abnormal glucose metabolism by oral glucose tolerance test (OGTT) in CF patients aged ≥10 years [1]. A small study suspected that CF patients have a high variation in glucose tolerance over time [2].

Therefore, we aimed to investigate in this short report the variability of glucose tolerance in a large cohort of CF patients. For the first time, a within-patient coefficient of variation (CV) for 2-hour blood glucose was calculated in CF.

## Materials and Methods

### Ethics statement

The ethical committees of Ulm University and Hannover Medical School approved the study, and patients or their parents provided written informed consent.

### Study subjects

Between 2001 and 2010, 1,778 CF patients aged 10 years or older were screened serially by OGTT in a multicenter screening program carried out by 43 German/Austrian specialized centers (Trial No. NCT00662714). All patients had a physician-based diagnosis of CF according to the latest German guidelines [3]. OGTTs were performed and interpreted in line with recommendations of the World Health Organization modified by the American Diabetes Association as described in detail previously [4], [5].

**Figure 1:**
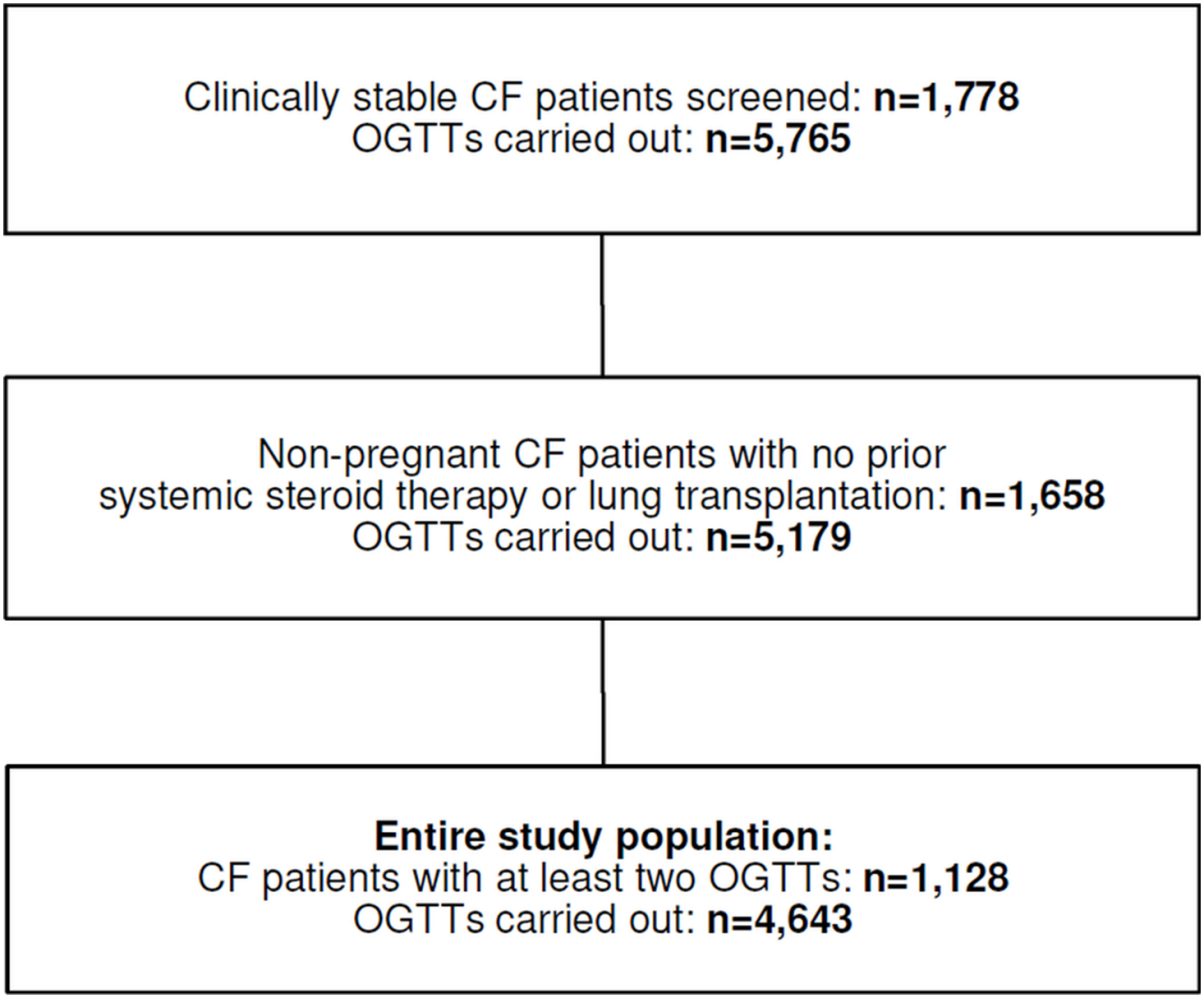
Selection of study population. Abbreviations: *CF* cystic fibrosis, *OGTT* oral glucose tolerance test.

In total, 5,765 OGTTs among clinically stable CF patients were carried out until the end of the study (Figure 1). As mentioned in reference [4], all patients screened had to be without symptoms of acute infections or exacerbations of chronic infections and should not have a reduced carbohydrate intake. Moreover, patients with respiratory failure (FEV1<40%) or with enteral tube feeding were excluded. In case of a confirmed diagnosis of CFRD or the use of anti-hyperglycemic treatment, patients were also excluded from screening.

For the present analysis, only OGTTs of non-pregnant patients with no prior systemic steroid therapy or lung transplantation were considered (Figure 1). If patients awaited lung transplantation, OGTTs were also excluded. Furthermore, at least two OGTTs had to be performed per patient (Figure 1). The final study population comprised 4,643 OGTTs of 1,128 CF patients. For each patient included, test results were classified as normal glucose tolerance (NGT), impaired fasting glucose (IFG), impaired glucose tolerance (IGT), IFG and IGT combined, or diabetic glucose tolerance (DGT). The detailed cut-offs are described in reference [4]. As there are some CF patients with isolated IFG [4] and the pathophysiology between IFG and IGT is at least in part different [6], we introduced an IFG group in this analysis. CFRD was defined as a diabetic OGTT confirmed by a consecutive OGTT in the DGT range [1]. Body mass index standard deviation score (BMI-SDS) was calculated using data from the German Health Interview and Examination Survey for Children and Adolescents (KiGGS study, [7]) as national reference. The KiGGS study was used as a reference in a number of previous publications by our group to calculate BMI-SDS in CF patients [4], [8], [9] and is the best currently available reference data for healthy German children. Data to calculate BMI-SDS on the basis of CF-specific anthropometric reference percentiles are currently not available.

### Statistical analysis

To analyze the variation in glucose tolerance, transition frequency from any one test to the subsequent test was studied. As there is no subsequent test for the last test in each patient, 3,515 pairs of consecutive OGTTs were available in the study population (the number of OGTT pairs therefore equals the total number of OGTTs minus the number of patients). For each pair of OGTTs, the first test was categorized in one of the five glucose tolerance classes (NGT, IFG only, IGT only, IFG+IGT, DGT) and the percentage of subsequent test results was calculated. Moreover, the variation in glucose tolerance is depicted in three individual patients who had seven consecutive OGTTs with at least one diabetic result. To assess the degree of variability of serially measured fasting and 2-hour blood glucose values in CF patients, within-patient CVs (defined as ratio between within-subject standard deviation and mean) were calculated and compared to the general population. CVs were calculated as they are normalized measures of the variability and independent of the mean level.

To evaluate gender or age differences in the deterioration of glucose tolerance from test to test, a logistic mixed regression model was applied. Deterioration of glucose metabolism was defined as worsening of glucose tolerance in a subsequent test compared to the pretest or as confirmation of a diabetic OGTT. Sex and age were entered as independent fixed effects. Age was categorized as 10–<15 years, 15–<20 years and ≥20 years. To account for repeated OGTTs per patient, random effects with first-order autoregressive covariance structure were used. Results are given as odds ratios (OR) with lower and upper confidence limit (CL).

Statistical analyses were performed with SAS 9.4. The level of significance was set at 0.05. To compare demographics between patients included and excluded, Kruskal-Wallis test was applied for continuous parameters and χ^2^-test for dichotomous parameters.

## Results

48.8% of patients included were female and 28.9% were >20 years. Median [Q_1_;Q_3_] (mean±SD) age at first OGTT was 15.5 [11.5; 21.5] (18.0±8.4) years and at last OGTT 18.6 [15.3; 25.4] (21.3±8.5) years. At baseline OGTT, the youngest patient aged 10.0 years and the oldest patient 63.9 years. On average, median (mean) duration between two consecutive OGTTs was 1.0 [0.7; 1.2] (1.1±0.7) year. A diabetic OGTT was repeated within a median (mean) time of 6.9 [4.9; 12.9] (9.1±6.2) weeks. As published previously [4], the median duration between two consecutive OGTTs in the DGT range (confirmed diagnosis of CFRD) was 8.1 [5.1; 27.9] weeks. All patients with suspected CFRD (i.e. one OGTT in the DGT range) at the end of the study were followed-up outside the context of the screening study. Each patient with a confirmed diagnosis of CFRD was invited to participate in a randomized controlled trial investigating the use of repaglinide in comparison to insulin therapy. CFRD patients not participating in the clinical trial received insulin therapy and CF-specific nutritional counseling as recommended by guidelines.

Baseline characteristics of the 650 patients excluded are depicted in table 1. Excluded patients were older and had on average a lower BMI-SDS.

**Table 1:** Demographics of patients included and patients excluded at baseline OGTT.

	Patients included	Patients excluded	p-value
Number, n	1,128	650	-
Age, years	15.5 [11.5; 21.5] (18.0±8.4)	17.4 [12.3; 25.2] (20.0±9.3)	<0.001
Female sex, %	48.8	45.1	NS
Weight, kg	47.8 [34.5; 57.8] (47.8±15.4)	50.2 [35.5; 59.0] (49.0±15.2)	0.04
Height, cm	160.0 [145.6; 170.0] (157.8±15.6)	162.0 [147.0; 172.0] (159.8±15.9)	0.008
BMI, kg/m^2^	18.4 [16.2; 20.4] (18.6±3.3)	18.6 [16.2; 20.7] (18.7±3.3)	NS
BMI-SDS	–0.76 [–1.42;–0.11] (–0.80±1.08)	–0.87 [–1.65;–0.18] (–0.99±1.26)	0.005

Data are given as median with quartiles (mean with standard deviation) or as percentage. Abbreviations: *BMI* body mass index, *SDS* standard deviation score, *NS* not significant.

**Figure 2:**
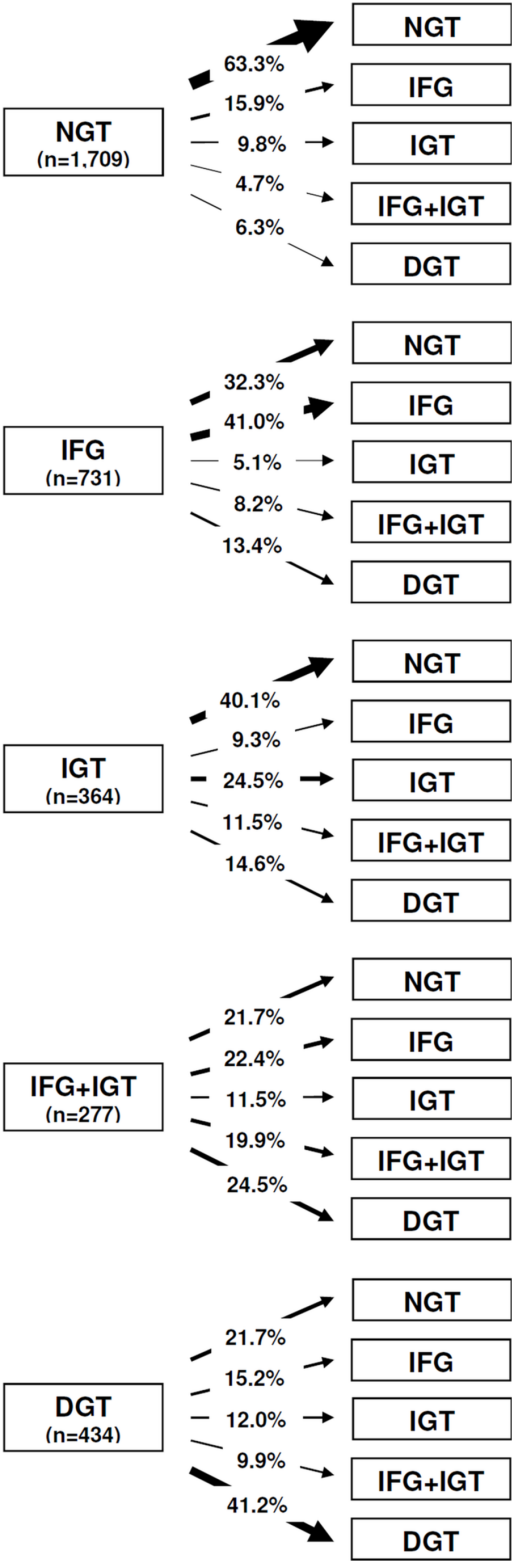
Transition from any one test to a subsequent test. In total, 3,515 pairs of consecutive OGTTs are summarized. For each pair, the first test was categorized in one of the five glucose tolerance classes (NGT, IFG only, IGT only, IFG+IGT, DGT). The figure shows the percentage of subsequent test results for each glucose tolerance category. The number in parentheses indicates the number of OGTTs available for analysis. Abbreviations: *NGT* normal glucose tolerance, *IFG* impaired fasting glucose, *IGT* impaired glucose tolerance, *DGT* diabetic glucose tolerance, *OGTT* oral glucose tolerance test.

Figure 2 displays the transition frequency from any one test to a subsequent test. For example, an OGTT in the DGT range was confirmed in 41.2% by the subsequent test.

Until the end of the screening study, a total of 136 CF patients revealed a confirmed diagnosis of CFRD i.e. two consecutive OGTTs revealing DGT. Despite the fact that two consecutive OGTTs in the DGT range are sufficient to diagnose CFRD, 54 out of the 136 patients had additional OGTTs. In 42.6% (n = 23) of patients, all subsequent tests were diabetic. In 20.4% (n = 11) of cases, at least one of the subsequent tests revealed NGT.

**Figure 3:**
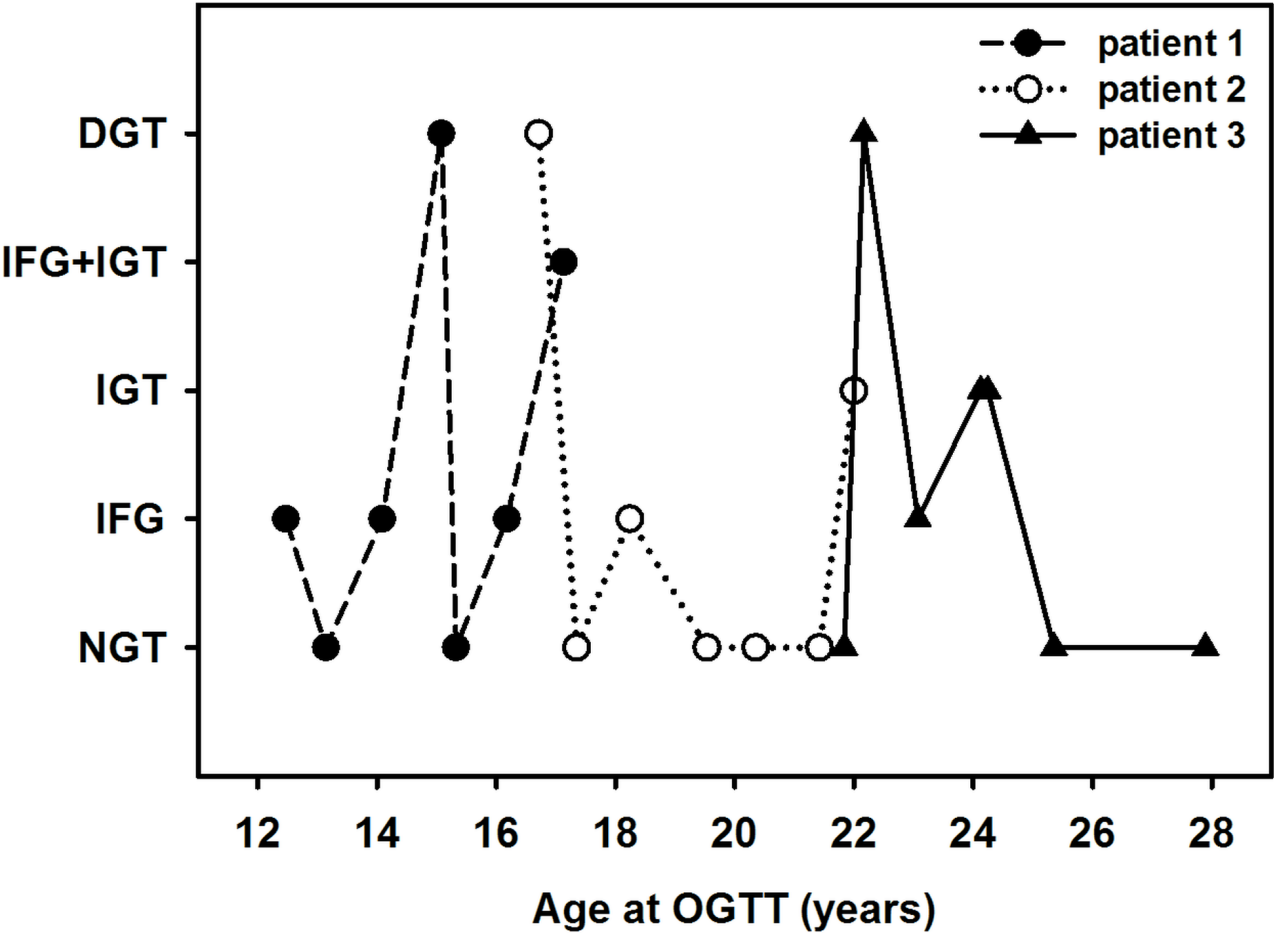
Variation of glucose tolerance in three patients of different age. Each patient had seven consecutive OGTTs with at least one diabetic result. Abbreviations: *DGT* diabetic glucose tolerance, *IFG* impaired fasting glucose, *IGT* impaired glucose tolerance, *NGT* normal glucose tolerance, *OGTT* oral glucose tolerance test.

Figure 3 indicates that independent of age, glucose tolerance varied highly from test to test in three individual patients.

Overall, in CF patients the average within-patient CV for fasting blood glucose values was 11.1% and for 2-hour blood glucose 25.3%. Analyzing patients with (n = 339) and without (n = 789) at least one OGTT in the DGT range separately, patients without an OGTT in the diabetic range had lower CVs for both fasting and 2-hour blood glucose values (fasting: 8.9 vs. 16.1%, p<0.001; 2-hour: 22.2 vs. 32.4%, p<0.001).

Logistic regression analysis revealed for girls a 1.18-fold [lower CL: 1.03; upper CL: 1.36] higher risk for the deterioration of glucose tolerance compared to boys (p = 0.015). In addition, patients aged 15–<20 years (OR: 1.23 [1.04; 1.47], p = 0.019) or ≥20 years (OR: 1.22 [1.03; 1.44], p = 0.024) had a higher risk than patients aged 10–<15 years. In 15 to <20 year old patients, risk was comparable to patients aged ≥20 years (OR: 1.01 [0.86; 1.19], p = 0.867).

Analyzing the influence of sex in the subgroup of older patients (>20 years at baseline OGTT; n = 326 patients with 1,276 OGTTs), revealed also an increased risk in females compared to males, even though the influence was no longer significant due to the lower number of patients studied (OR: 1.23 [0.92; 1.63], p = 0.162).

## Discussion

Our analysis among asymptomatic CF patients revealed a high variability in glucose tolerance over time, even though patients without DGT seem to be more stable with respect to glucose tolerance. Compared to the general population [10]–[12], overall within-patient variability of 2-hour blood glucose is 1.5 to 1.8-fold higher in CF. The NHANES III Second Examination revealed a within-patient CV of 16.6% for 2-hour blood glucose [12] and the Hoorn study of 16.7% [11]. In a general practice [10], a within-patient CV of 14.3% was observed. Fluctuating levels of insulin resistance due to acute and chronic infections or CF-related inflammatory cytokines may explain the highly variable glucose tolerance in CF.

The fluctuation in glucose tolerance observed in this study implicates regular control of glucose metabolism in CF. Future guidelines should provide recommendations regarding an adequate time frame for repeating OGTTs in patients with abnormal glucose tolerance as well as for the confirmation interval of a diabetic test result. As outlined by the study protocol and described previously [4], a diabetic OGTT had to be repeated within 4 to 6 weeks in the present study and an OGTT revealing IFG or IGT after 6 months. According to guidelines, all patients with a confirmed CFRD diagnosis or with a diabetic OGTT and classical symptoms of diabetes should receive insulin treatment immediately [1]. Oral antidiabetic agents are not advised until sufficient data become available regarding their efficiency.

Reasons for performing additional OGTTs in patients with already confirmed CFRD are unknown. Maybe patients requested an additional OGTT in the context of intensifying therapy. Especially, asymptomatic CF patients often have problems starting insulin treatment besides numerous CF-related therapies. Another possibility might be that patients with a confirmed CFRD diagnosis were not totally clinically stable at the time of the confirmation test and the medical team therefore decided to repeat the test at a later time point. As OGTT results depend also on the supplementation of pancreatic enzymes, the medical team may have repeated the confirmation test after adjusting pancreatic enzyme substitution.

Although the OGTT revealed a high degree of within-patient variance in CF, it is the best screening tool for abnormalities in glucose tolerance currently available. Fasting plasma glucose, hemoglobin A1c or other classical methods applicable for type 1 or type 2 diabetes are not recommended in CF [1]. In the future, continuous glucose monitoring or standardized test meals might be a useful alternative to OGTT. However, there are no reference values for continuous glucose monitoring so far. To minimize the variability of OGTTs in CF patients, CF-adapted standardization might be one possibility. At the moment, criteria for the performance and interpretation of OGTT are identical to other diabetes types. However, pancreatic enzyme substitution and the grade of inflammation (e.g. C-reactive protein measurement) should be considered before performing an OGTT in CF, and when interpreting OGTT results. More advanced interpretation criteria that take into account dynamics in oral glucose challenge in CF patients might be appropriate. As CFRD is less common compared to the more frequent type 1 or type 2 diabetes, other measures of ß-cell function (e.g. C-peptide) or calculation of indices for insulin secretion and sensitivity might be practicable in clinical settings that may result in better assessment of glucose tolerance in CF patients.

The higher risk for deterioration of glucose tolerance in girls confirms previous studies indicating an earlier onset and a higher prevalence of CFRD in females [4], [13], [14]. The lower risk in patients younger than 15 years compared to older patients might be explained by the rare occurrence of CFRD in patients <10 years and the increasing prevalence of abnormal glucose tolerance with age [4], [15], [16].

Selection biases might be one limitation of the present study. Not all centers or CF patients participated, although all German/Austrian medical facilities providing care for CF patients were invited for participation in written form. Moreover, some CF-related therapies (e.g. steroids, lung transplantation) led to exclusion of patients and the complexity of CF itself made study participation often difficult. The higher age and the lower BMI-SDS of patients excluded may point to a more severe progression of the disease compared to patients included. Exclusion criterion for the majority of the 650 patients not analyzed was the availability of solely one OGTT. This might be due to termination of the study or occurrence of exclusion criteria after the first OGTT. Moreover, each patient could withdraw from the study at any time.

In summary, in the asymptomatic CF patients studied, one diabetic OGTT seems not sufficient to diagnose CFRD due to the existing high variability of glucose tolerance. A normal glucose tolerance after a confirmed CFRD diagnosis (i.e. two consecutive OGTTs revealing DGT) cannot be excluded. Despite improvement of glucose tolerance, these patients are classified as diabetic [1], although this labelling may be contra-intuitive for patients and physicians. It is still a debate whether solely a persistently diabetic glucose tolerance is associated with an increased risk of clinical decline in CF or even a pre-diabetic condition.
